# Sustainable Alkaline Hydrolysis of Polyester Fabric at Low Temperature

**DOI:** 10.3390/ma15041530

**Published:** 2022-02-18

**Authors:** Ivana Čorak, Anita Tarbuk, Dragan Đorđević, Ksenija Višić, Lea Botteri

**Affiliations:** 1 Department of Textile Chemistry and Ecology, University of Zagreb Faculty of Textile Technology, Prilaz Baruna Filipovića 28a, HR-10000 Zagreb, Croatia; ivana.corak@ttf.unizg.hr (I.Č.); ksenija.visic@ttf.unizg.hr (K.V.); lea.botteri@ttf.unizg.hr (L.B.); 2 Textile Department, Faculty of Technology, University of Niš, Bulevar Oslobođenja 124, 16000 Leskovac, Serbia; drdrag64@gmail.com

**Keywords:** PET (poly(ethylene terephthalate)) fabric, alkaline hydrolysis, process sustainability, PET dissolution kinetic model

## Abstract

High crystallinity leads to low hydrophilicity of fabric made of (poly(ethylene terephthalate)) fibers (PET) causing problems in finishing, washing, and dyeing processes. To improve these properties, the surface of PET fibers is usually modified by hydrolysis. Alkaline hydrolysis is a conventional process usually performed at a temperature higher than 100 °C for more than 1 h. However, the use of strong alkali and high processing temperatures (>100 °C) can lead to fabric damage and a negative impact on the environment. Therefore, in this paper, the possibility of hydrolysis of the PET fibers in the fabric in a sustainable, energy-efficient process was researched. The influence of low temperature (60–100 °C) and an accelerator (a cationic surfactant HDTMAC) to PET alkaline hydrolysis was studied through weight loss, the loss in breaking force, and fiber morphology. The kinetics of PET dissolution in 1.5 mol cm^−3^ NaOH at low temperature with and without the addition of HDTMAC was determined and the activation energy was calculated according to the theoretical model. It has been confirmed that PET hydrolysis can be carried out in 1.5 mol cm^−3^ NaOH with the addition of HDTMAC as an accelerator at 80 °C for 10 min. This process is more economically and energetically acceptable than the conventional process, and is therefore more sustainable.

## 1. Introduction

Polyester fiber is, by definition, ‘a fiber composed of linear macromolecules having in the chain at least 85% by mass of an ester of a diol and terephthalic acid’ [1]. All types of polyester polymers for fibers are thermoplastic and can be used for the melt spinning process resulting in requested properties for certain usages (i.e., filaments, microfibers, anti-pilling, antistatic, excellent mechanical properties, chemical resistance, etc.). Regardless of properties achieved during spinning, polyester fibers have a relatively high degree of crystallinity, and therefore low absorptions of water and moisture from the air (regain only 0.4%). Poly(ethylene terephthalate) (PET) is not only the most widely used polyester fiber but the most used synthetic fiber as well. It can be also used for the production of foils and films, in electrical engineering and electronics as a construction material, for packaging (beverage bottles, bags), lighting, automotive products, sports equipment, etc. [2,3]. 

**Figure 1:**
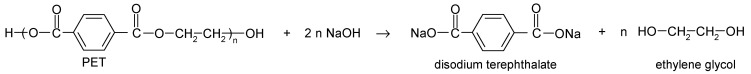
PET hydrolysis.

The low hydrophilicity of fabrics made from PET fibers causes problems in finishing, washing, and dyeing processes, and can result in the formation of piling, accumulation of static electricity and attraction of oil soils, and low adhesion to plastics and rubber. To improve these properties, the fibers can be copolymerized, blended or designed to incorporate hydrophilic compounds. Usually, however, the surface of the PET fibers is modified. A PET surface can be modified by coating, grafting, plasma, UV laser, and chemical reactions such as hydrolysis and aminolysis [3,4,5,6,7,8,9,10,11,12,13,14,15,16,17,18,19,20,21,22,23,24,25,26,27,28,29,30,31,32,33,34,35,36,37,38,39,40,41,42,43,44,45,46,47,48,49]. Plasma surface modification can operate in two directions. The first includes chain scission, cross-linking, and intra- and inter- molecular reaction of chains, and the second a reaction with chemicals. This has advantages compared to conventional surface modifications regarding the reduction of pollutants (no water or chemical required). However, it is still a very expensive method for many industries, especially if helium is applied [14,29,30,31]. Surface grafting includes radical initiated reactions by chemicals or energy sources such as UV light and plasma. It can lead to better hydrophilicity or hydrophobicity (depending on the chemicals used) [14]. Ester-amine interchange reactions have also been researched for surface functionalization. Aminolysis with ethylenediamine for a short time in ambient conditions results in the creation of both amine and carboxylic acid functional groups on the polyester fiber surface, which provides a possibility for better finishing effects [18,32,33,34]. Hydrolysis can be performed with alkaline and enzymes [3,4,5,6,7,8,9,10,11,12,13,14,15,16,17,18,19,20,21,22,23,24,25,26,27,28,29,35,36,37,38,39,40,41,42,43,44,45,46,47,48,49]. The mechanism of hydrolysis is shown in Figure 1.

In alkaline hydrolysis, the hydroxyl ions from alkali attack the electron-deficient carbonyl carbons along the PET main chain, causing scissions at the ester linkages and the production of hydroxyl and carboxylate end groups [3,14,18,29]. It results in physical and chemical changes in the fiber surface with the formation of small craters and an increasing surface area with extra functional groups. Hydrolyzed fabrics have better wettability and dyeability and a silk-like appearance and luster. However, hydrolysis also results in pitting corrosion (etching) with a negative impact on fabric strength [3,9,10,11,12,13,14,15,22,23,24,25,26,29,39]. Furthermore, it requires a high amount of sodium hydroxide, high energy, and water consumption. Alkaline hydrolysis is usually performed in 4–20% KOH and NaOH at temperatures higher than 100 °C (130–140 °C) for more than 1–2 h. The alkaline hydrolysis performed in addition to different cationic surfactants and polymers [6,7,8,9,10,11,12,13,14,15,19,20,21,22,23,24,25,26] can accelerate this process. It was found that some cationic surfactants and polymers accelerate the process (but not all). For example, Gawish et al. [6,7,8,9] studied a series of cationic surfactants synthesized from tetramethyl ethylenediamine with the quaternizing agents cetyl bromide, benzyl chloride, or 2-chloroethanol and a series of cationic polymers of different chain length, polydimethylaminoethyl methacrylate quaternized with cetyl bromide or combinations of cetyl bromide and benzyl chloride or cetyl bromide and 2-chloroethanol, and found that cetylethylmetacrylatedimethylammonium bromide and oleyl-*bis*(2-hydroxyethyl)cetylammonium bromide accelerated hydrolysis from 6 h to 20 min under the same process conditions (100–130 °C). They also synthesized cationic surfactants based on diethanolamine and alkyl halides such as cetyl bromide with epichlorohydrin as a quaternizing agent. However, prior to hydrolysis, the polyester was pretreated with solvents (i.e., tetrachloroethane and tetrachloroethylene). Jemaitaitis et al. [20] compared tetraethylammonium bromide, benzyltriethylammonium chloride, poly(diallyldimethylammonium chloride), and the diethyldimethylammonium derivative of a benzenesulfonate polyglycol ester as accelerators and found that the cationic polymers had a greater effect due to the hydrophobic interaction with the fiber surface. Grancarić et al. [10,11,12,13,19] studied commercial surfactants anionic, nonionic, and cationic (as well as an epihalohydrin) and found that Lyogen BPN, a fatty acid amineamide, and an epihalohydrin, 3-chloro-2-hydroxypropyl)trimethyl ammonium chloride, both accelerate hydrolysis. In the same research group, Pušić, Tarbuk et al. [50,51,52,53,54] studied adsorption and desorption of ionic surfactants under the influence of fiber composition and surfactant ionogenity as well as variation of hydrophobic chain length and hydrophilic groups in the molecule. Dodecyltrimethylammonium bromide (DDTMAB), tetradecyltrimethylammonium bromide (TDTMAB), hexadecyltrimethylammonium bromide (HDTMAB), hexadecylpyridinium chloride (HDPC) and hexadecyltrimethylammonium chloride (HDTMAC) were studied in detail. It was proven that the cationic surfactants are attracted by electrostatic interactions with the negatively charged fiber surface and that their adsorption is enhanced by the length of the alkyl chain. The adsorbed amount on PET depends on the presence of hydrophobic interactions between the cationic surfactants and the PET fibers. Therefore, the surfactants with longer chains adsorb better on PET. The adsorption of HDTMAC and HDTMAB is similar, and HDPC (CPC) is lower than HDTMAC since the aromatic ring in the pyridinium group decreases the adsorption but increases the stability of the already adsorbed surfactant in the fiber [50]. Since HDTMAC and HDTMAB (CTAB) show similar adsorption (due to the same alkyl chain), HDTMAC was selected as accelerator for alkaline hydrolysis due to its better water solubility.

Recently, ionic liquids have been used to reduce the weight of polyester, up to 20% [22,23,24,25,26]. Dong et al. [24] investigated alkaline hydrolysis in the presence of different 1-alkyl-3-methylimidazolium bromine ionic liquids (ILs) with different numbers of C-atoms (i.e., CnMImBr (*n* = 8, 12, 14, 16)) and compared them to cetyltrimethylammonium bromide (CTAB) as accelerators. It was found that CnMImBr with a long carbon chain can be used as a novel accelerator, which can be explained by the higher adsorption capacity of polyester for the ILs or cationic surfactants with longer carbon chains. Liu et al. [25] investigated PET hydrolysis using the ionic liquid 1-n-butyl-3-methylimidazolium chloride as a solvent and the acid-functionalized ionic liquid 1-methyl-3-(3-sulfopropyl)-imidazolium hydrogen sulfate as catalyst and found that complete degradation occurred after 4.5 h at 170 °C. Musale et al. [26] treated the PET fabric with aqueous and methanolic NaOH solutions in the presence of a quaternary ammonium compound, cetyltrimethyl ammonium bromide (CTAB), and the ionic liquid 1-butyl-3-methylimidazolium chloride ([BMIM]Cl). [BMIM]Cl proved to be more effective than CTAB and the weight loss in aqueous NaOH was less than in methanolic NaOH. Cao et al. [21,22,23] used cetylpyridinium chloride (CPC) as an accelerator to perform alkaline de-weighting and dyeing of polyester fabric as a one-step process. They also studied ionic liquids as well as alkyl imidazolium gemini ionic liquids as accelerants for alkali de-weighting of polyester fabrics, and compared it with conventional cationic surfactants cetyltrimethyl ammonium chloride or 1227 (dodecyldimethylbenzylammonium chloride), respectively. It was found that the ionic liquids and alkyl imidazolium gemini ionic liquid can be used for alkali de-weighting. Due to the irreversibility of the reaction, it is necessary to control the process of alkaline hydrolysis if this process is to result in de-weighting or complete degradation. The optimal weight loss is between 10 and 24%, and the breaking force (mechanical damage) can be up to 35%. In harsher processing conditions, cracks and often holes occur, suggesting fabric damage [6,7,8,9,10,11,12,13,14,15,16,17,18]. By controlling the processing time, it can be used to engineer sub-micro roughness (also called alkali etching) on a fiber surface to make superhydrophobic, self-cleaning textiles [45,46,47,48,49]. 

The use of strong alkali and high processing temperatures can lead to material damage but also to environmental pollution. Environmentally friendly enzymes have been investigated in recent years as an alternative to alkali. For the hydrolysis of PET, cutinases, esterases, lipases, laccases, and polyesterases (serine esterase) can be used [3,12,35,36,37,38,39,40,41,42,43,44]. For the difference of alkali hydrolysis, due to the size of enzymes, they can only act on the surface of the fiber, not making crates or fiber damage. However, its application has been investigated mainly on films and foils and not on heterogeneous textiles [3,35,36,37,38,39,40,41,42,43,44].

The European Technology Platform (ETP) launched seven strategic programs among including sustainable chemistry related to the design, development, and application of environmentally friendly products and energy-efficient processes. In accordance with this strategic guideline, in this work the possibility of alkaline hydrolysis of the PET fibers in the fabric in a sustainable, energy-efficient process was researched. For this purpose, PET alkaline hydrolysis was performed at low temperatures (60–100 °C) with and without accelerator, a cationic surfactant HDTMAC, monitoring the weight loss, breaking force, mechanical damage, and fiber morphology. The polyester dissolution kinetic model was calculated from the weight loss results to determine whether alkaline hydrolysis of PET fabric is possible at low temperatures.

## 2. Materials and Methods

### 2.1. Material

A commercial fabric in satin weave fabric (Belira, Banja Luka, Bosnia and Herzegovina) produced from 100% poly(ethylene-terephthalate) PET fibers was used. It was made of multifilament yarns (16f) of warp 38 cm^−1^ and weft 29 cm^−1^ density and the fineness 50 dtex in both warp and weft (i.e., fiber radius 9 μm) with a molar mass of PET unit 192 gmol^−1^; fabric mass per unit area 60 gm^−2^; and having been stabilized with hot air.

### 2.2. Modification 

**Figure 2:**
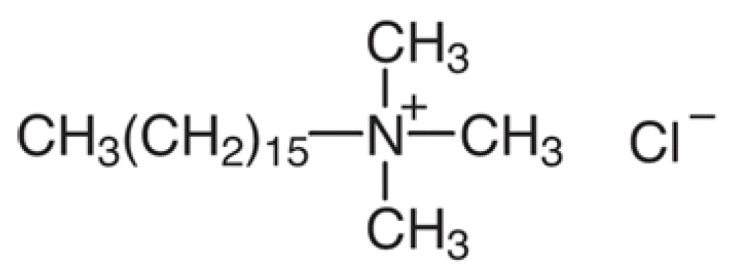
Hexadecyltrimethylammonium chloride (HDTMAC).

Alkali hydrolysis was performed with 1.5 mol cm^−3^ sodium hydroxide (NaOH, Sigma-Aldrich Co.-Merck KGaA, Darmstadt, Germany) without and with the accelerator, a cationic surfactant Hexadecyltrimethylammonium chloride (HDTMAC, 25% aqueous solution, Merck KGaA, Darmstadt, Germany), Figure 2. 

Fabric modification was made by a batch wise method in stainless-steel bowls of instrument Linitest, (Original-Hanau, Hanau, Germany) with LR 1:50, with the variation of temperature and time. The process temperature varied from 60, 70, 80, and 90 to 100 °C. The process time was 15, 30, 45, and 60 min (with an additional 5 and 10 min if the accelerator was added). Hydrolyzed samples were rinsed in hot water for removal of oligomers, then rinsed in warm and cold distilled water, neutralized with 5% acetic acid (CH_3_COOH), and again rinsed with distilled water to neutral pH. Samples were dried at 105 °C in dryer ST-01/02 (Instrumentaria, Zagreb, Croatia). 

### 2.3. Characterization Methods

Mass per unit area (*m*) in gm^−2^ was determined according to ISO 3801:1977 Textiles–Woven fabrics-Determination of mass per unit length and mass per unit area, using an analytical balance, model ALJ 220-5DNM (KERN & Sohn GmbH, Balingen, Germany) having a measurement accuracy of 0.0001 g. Weight loss (Δ*m*) in % was calculated on an absolute dry sample.

Breaking force (*F*) in N and elongation (ε) in % were determined according to ISO 13934-1:2013 Textiles-Tensile properties of fabrics-Part 1: Determination of maximum force and elongation at maximum force using the strip method on dynamometer Tensolab (Mesdan S.p.A., Puegnago del Garda, Italy). From the breaking force the mechanical damage (Um) i.e., loss of breaking force was calculated according to ISO 4312:1989 Surface active agents—Evaluation of certain effects of laundering—Methods of analysis and test for unsoiled cotton control cloth:

(1) $$U_{m}=\frac{F_{0}-F}{F_{0}}\cdot 100\;[\%]\;$$

where *U_m_* is the mechanical damage in %, *F*_0_ is the breaking force of untreated and *F* is the breaking force of the alkali hydrolyzed PET fabric in N. 

The hydrophilicity was tested according to AATCC TM 79-2014 *Absorbency of Textiles*, (i.e., the drop test).

The surface morphology of PET fibers in selected fabrics was analyzed from micrographs taken on a scanning electron microscope (SEM) FE-SEM, Mira II, LMU (Tescan, Brno, Czech Republic) with a magnification of 3000×. The fabrics were coated with a thin layer of chromium for 120 s.

### 2.4. Model for Kinetics of Polyester Dissolution

The theoretical model developed by Kallay and Grancaric in 1990 [11,12,13] was used to describe the kinetics of polyester dissolution in alkaline solutions. According to the model, PET hydrolysis in alkali solution can be expressed as:

PET + 2 OH^−^ → disodium terephtalate + ethylene glycol (2)

and the dissolution rate as:

(3) $$\frac{dn}{dt}=-kAc^{h}$$

where *n* is the amount in moles of undissolved PET units, *t* is time, *A* is the active surface area, *c* is the concentration of OH^-^ ions in NaOH, *k* is the rate constant of hydrolysis and *h* is the order of reaction with respect to OH^-^ ions. Equation (2) suggests that every terephthalate unit requires two moles of NaOH for the complete reaction, and the weight loss is equivalent to the quantity of alkali consumed in the reaction. During the process, the fiber surface area and the ion concentration both reduce, respectively. According to the model, considering both reactants, the reaction is of the first order (h = 1). The following expression can be obtained:

(4) $$F_{t}=-\frac{kV_{m}\sqrt{n_{0}}}{r_{0}}t+F_{0}$$

where *n*_0_ is the initial amount of PET, *r*_0_ is the initial radius of fibers, *V_m_* is the molar volume of PET. Function *F* (at time *t*) and *F*_0_ (at time *t* = 0) are defined for the initial excess of base, *Vc*_0_ > *2 n*_0_ (*V* is the volume of the base solution) through the amounts of PET at time *t*, *n_t_*, as follows:

(5) $$Ft=\sqrt{\frac{V}{2c_{0}-4n_{0}}}\cdot \arctan\sqrt{\frac{2nt}{c_{0}V-2n_{0}}}$$

For interpretation of the data, the function *F* should be plotted vs. time *t* and the slope of linear yields the rate constant *k*, from which the mass of undissolved PET can be calculated. The dependency of dissolution constant rate on temperature can be expressed by the Arrhenius equation:

(6) $$k=B\;e^{\frac{-Ea}{RT}}$$

where *B* is the pre-exponential factor (i.e., pre-exponential collision frequency factor), *R* is the gas constant, and *T* is the thermodynamic temperature.

## 3. Results and Discussion

### 3.1. Optimization of Energy Efficient Process

**Figure 3:**
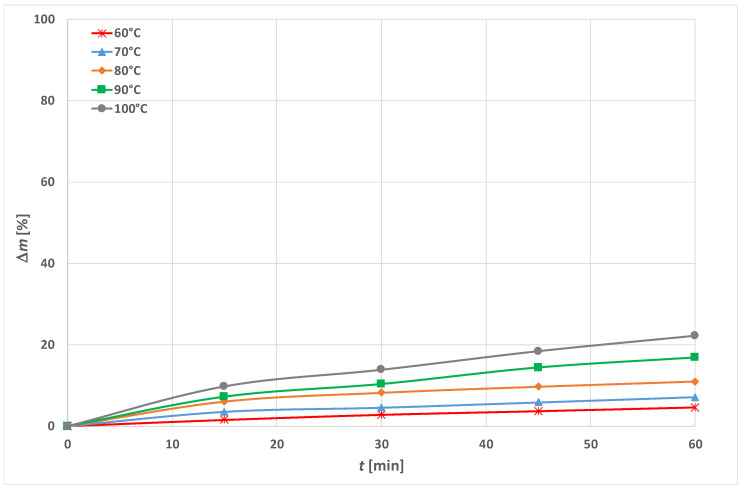
Dissolution of PET fibers in 1.5 mol cm^−3^ NaOH at temperature range 60 to 100 °C expressed as weight loss (Δ*m*).

**Figure 4:**
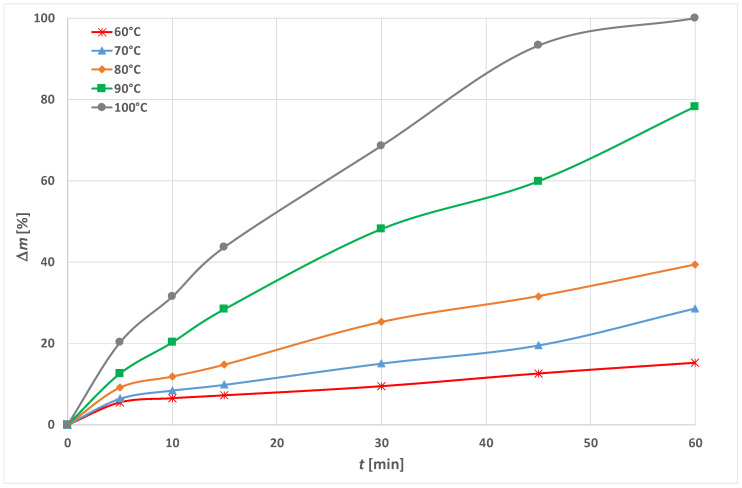
Dissolution of PET fibers in 1.5 mol cm^−3^ NaOH with addition of 1 gcm^−3^ HDTMAC at temperature range 60 to 100 °C expressed as weight loss (Δ*m*).

**Figure 5:**
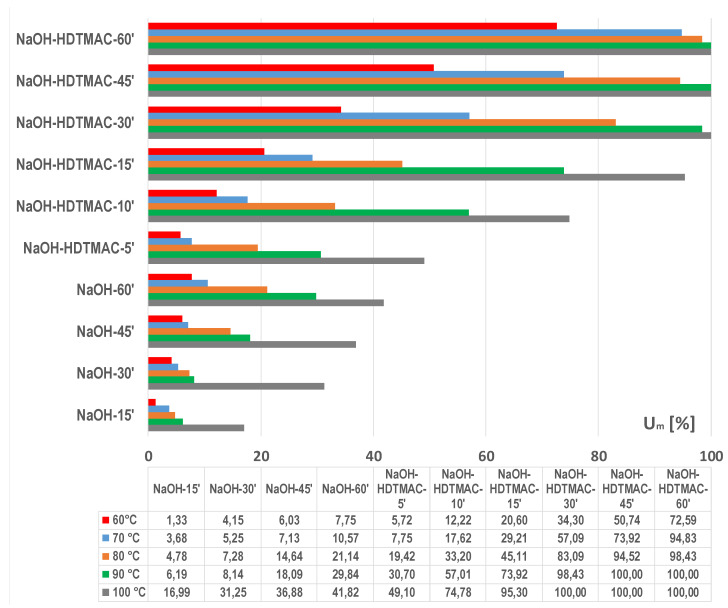
The PET fabric damage after alkali hydrolysis in 1.5 mol cm^−3^ NaOH without and with addition of HDTMAC at temperature range 60 to 100 °C expressed as mechanical damage (*U_m_*).

**Figure 6:**
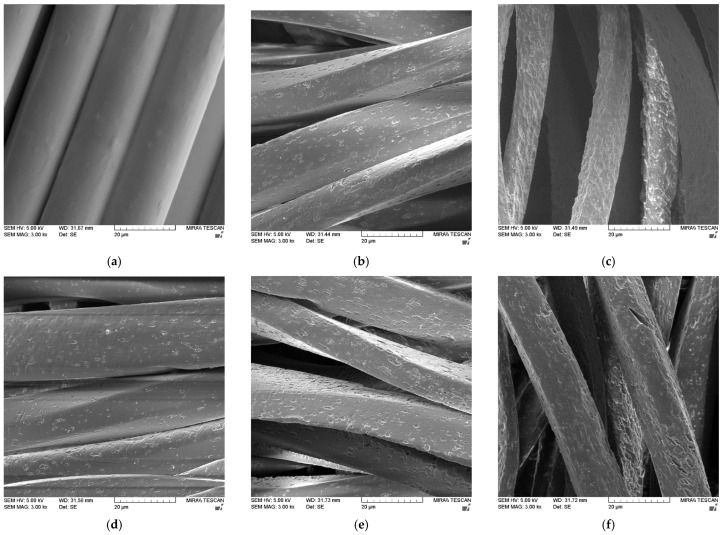

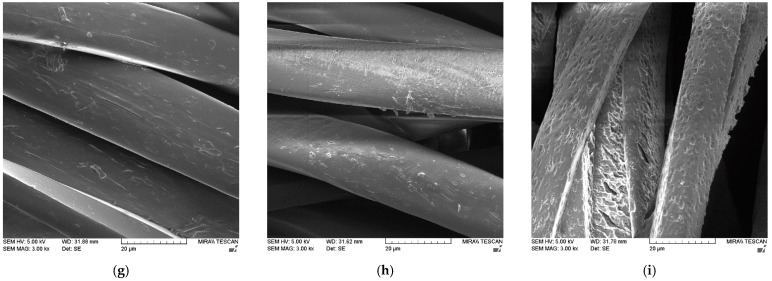
SEM micrographs of PET fibers in fabric at magnification 3000×: (**a**) untreated; (**b**) NaOH + HDTMAC-100 °C–5′; (**c**) NaOH + HDTMAC–100 °C–30′; (**d**) NaOH + HDTMAC–80 °C–10′; (**e**) NaOH + HDTMAC–80 °C–30′; (**f**) NaOH + HDTMAC–80 °C–60′; (**g**) NaOH + HDTMAC–60 °C–30′; (**h**) NaOH + HDTMAC–70 °C–30′; and (**i**) NaOH + HDTMAC–90 °C–30′.

In this paper, the influence of temperature on PET alkaline hydrolysis, with and without the addition of an accelerator (a cationic surfactant HDTMAC) was researched. The results of the weight loss (Δ*m*) of PET fabrics after hydrolysis in just 1.5 mol cm^−3^ NaOH are shown in Figure 3 and with the addition of 1 gcm^−3^ HDTMAC in Figure 4. The breaking force and elongation are presented in Table 1 and Table 2, and the calculated mechanical damage (*U_m_*) is shown in Figure 5. Fabric absorbency results are presented in Table 3. The characteristic morphology of PET fibers in fabrics after the hydrolysis for representative processes considering the selected temperature and time is shown in Figure 6 on SEM micrographs. 

Conventional alkaline hydrolysis, carried out for 60 min at 100 °C, results in a weight loss of 22.12%. The addition of the 1 g cm^−3^ of cationic surfactant HDTMAC at 100 °C, the reaction is accelerated, so in 5 min the weight loss is similar (i.e., 20.22%). The reaction time is reduced, which is efficient, but still energetically inefficient. Increasing the processing time results in increasing weight loss, while at 60 min the samples dissolves completely. Lowering the temperature to 60 °C without adding accelerator has no effect (Figure 3). If HDTMAC is added, and the time is increased, a similar result of 20% weight loss can be obtained at a lower temperature (Figure 4), i.e., at 90 °C in 10 min, at 80 °C in 20 min, and at 70 °C in 45 min. 

Simultaneously with weight loss, there is a loss of breaking force due to alkali etching of the PET fibers with alkali. Alkaline hydrolysis causes significant loss of breaking force, indicating mechanical damage of fabric for further processing. The acceptable loss of breaking force after processing is 20–40% to maintain wear properties [3,6,7,8,9,10,11,12,13,14,15,16,17,41].

From Table 1 and Figure 5, it can be noticed that hydrolyzed PET fabric in NaOH for 60 min at 100 °C has a loss of 41.82%, which is on the upper limit. For this reason, PET hydrolysis can be controlled to affect fabrics only as much as necessary (i.e., only surface hydrolysis) in order to improve water wettability without changes to the overall fiber geometry or extensive hydrolysis, resulting in damage to the constituent polymer. These results correlate to previous research on alkaline hydrolysis [3,4,5,6,7,8,9,10,11,12,13,14,15,16,17,18,47,48].

However, the results when HDTMAC is used as an accelerator show a similar phenomenon as when other cationic surfactants are used, but the dissolution of PET fiber occurs much faster. For example, to achieve the same effect at 100 °C 15 min were required when using Lyogen BPN (fatty acid amino amide by Sandoz), 45 min when using 3-chloro-2-hydroxylpropyltrimethyl ammonium chloride, and 65 min when using Tinegal PAC (quaternary ammonium compound by Ciba) as an accelerator [12,19,55,56,57,58]. Comparing the results to the ones obtained with CPC [21] weight loss is much higher with HDTMAC. The alkali concentration is lower, but also the presence of the aromatic ring in the pyridinium group reduces the surfactant adsorption [59]. The results obtained with CTAB (cetyl trimethylamonium bromide) in terms of weight loss are similar to those obtained with HDTMAC at high temperatures when CTAB was used in NaOH with lower concentration (10 g cm^−3^) at 95 °C [24]. Considering that previous researches using cationic surfactants as accelerators were at temperatures above 90 °C. Similar results for weight loss at 60 °C using HDTMAC as when CTAB was used as an accelerator were found when CTAB was used with a much higher concentration of NaOH (10%) [26]. Additionally, at that concentration ionic liquid C_16_MImBr showed same phenomenon [24].

The addition of HDTMAC as an accelerator causes the highest loss of breaking force. In 5 min the loss of fabric breaking force is 49.1%, and after 15 min the loss is greater than 95%. All treatments that have resulted in weight loss of 20% at lower temperatures show high mechanical damage as well. Therefore, a shorter time and lower weight loss should be considered. 

From the results shown in Figure 3, Figure 4 and Figure 5 it can be seen that alkaline hydrolysis at 90 °C without the addition of an accelerator for 60 min leads to a fabric weight loss of 16.76%. The addition of HDTMAC shows a weight loss of 12.56% at 5 min. Increasing the processing time leads to an increase of weight loss. The fabric treated for 15 min has a weight loss of 24.38% which exceeds the desired weight loss of 10 to 20%. Further increase of the processing time leads to even greater losses (78.12% for 60 min of processing).

By reducing the temperature to 80 °C, the alkaline hydrolyzed fabric without the addition of an accelerator for 60 min of processing has a weight loss of 10.94%. With the addition of the accelerator, almost the same weight loss of 9.11% is achieved in 5 min, and after 15 min the weight loss is 14.75%, which is within the desired weight loss range (10–20%).

The alkaline hydrolysis at 60 and 70 °C without an accelerator do not result in desired weight loss of 10% not even after 60 min. For 60 °C, the weight loss is 4.55% and at 70 °C it is 7.09%, respectively. With the addition of the accelerator, weight loss higher than 10% is obtained after 30 min at 70 °C (15.03%), and at 60 °C in 45 min (12.55%). 

By reducing the processing temperature, there is less weight loss, and therefore the loss in breaking force as well (Table 1, Figure 5). However, the PET fabric treated with the addition of HDTMAC at 90 °C for a period of 5 min recorded a limiting drop of breaking force of 30%, while the breaking force after 60 min could not be determined. 

For the difference of hydrolysis at 90 °C, at 80 °C the satisfactory strength results were obtained. Treatment of 5 min results in loss of 19.42%, and 10 min 33.20%. At lower temperatures 60 and 70 °C loss in strength is lower but the treatment is not satisfactory.

The weight loss and loss in breaking force are accompanied by a decrease in breaking elongation as well (Table 2). The elongation of the untreated sample is 34.200%. Treatment in NaOH results in less weight loss than when the accelerator was applied, and therefore the decreased elongation at the break is less. For example, after alkaline hydrolysis for 5 min at 100 °C in NaOH elongation is 33.240% while when HDTMAC is added, it is 25.781%. Considering the temperature, the elongation decrease is analogous to the weight loss (i.e., 10 min at 90 °C or 15 min at 70 °C). 

In regard to acceptable weight loss and fabric damage, the treatment at 80 °C for 10 min seems to be the optimal one. At a lower temperature the expected effect is not achieved, while at a longer process time the fabric is damaged too much. SEM micrographs shown in Figure 6 confirms this conclusion. 

The surface of PET fibers in fabrics before and after alkaline hydrolysis was analyzed by scanning electron microscopy (SEM). From the SEM micrographs, it can be seen that the fibers in the untreated PET fabric have a smooth surface (Figure 6a). 

In all alkaline hydrolyzed PET, fabrics can be seen that hydrolysis takes place on the fiber surface. The reason for this lies in the extremely nonpolar properties of PET, so that strongly ionized components, such as NaOH solution, cannot diffuse into the bulk. As a result, piling and craters appear on the surface. They appear in the transverse direction of the fiber axis and are caused by the breaking of chains in the amorphous region. Shallow craters which are only clearly visible on the surface, confirm that the modification was carried out under optimal conditions. 

PET fabrics have a similar micrograph of the fiber surface under the proposed optimal processing conditions-with the addition of HDTMAC for 5 min at 100 °C and for 10 min at 80 °C (Figure 6b,d). Deeper craters and holes in the transverse direction of the fibers are present on fibers in Figure 6c,f,i, suggesting significant damage while Figure 6e shows a similar result but less damaged fibers. This visible damage correlate with a large weight loss and a high loss of breaking force of those samples. On the other hand, Figure 6g,h show an insufficient effect of hydrolysis. Alkaline hydrolysis at low temperature for 30 min still keeps the surface smoother and correlates with less weight and strength loss. 

The hydrophilicity of polyester fabric after surface modifications was researched. The quick drop test was performed to determine the change in PET fabric absorbency in regard to untreated PET fabric. The results are presented in Table 3. 

In the drop test, the time required for the specular reflection of the water drop to disappear is measured and recorded. From the results presented in Table 3 can be seen that untreated PET fabric has a wetting time of 12 s. However, it has been observed that this fabric does not absorb the water drop, but it spreads very fast on warp and weft by capillary forces. Due to the change of the surface by hydrolysis, new hydroxyl and carboxyl groups are formed, and the surface area is increased so that the absorption capacity for water molecules is possible. Therefore, all hydrolyzed surfaces have better wetting properties [14,60]. According to the test, very good absorbency has been achieved, and excellent absorbency has been obtained for alkaline hydrolyzed surfaces with the accelerator at 80 °C (0 < t < 6s). 

### 3.2. Model for Kinetics of PET Fabric Dissolution with HDTMAC as an Accelerator

The experimental results of PET fabric weight loss in alkaline hydrolysis without and with the addition of HDTMAC as accelerator are given in Figure 3 and Figure 4. As can be seen, alkaline hydrolysis depends on temperature and the addition of cationic surfactant. It is non-linear and proceeds faster at higher temperatures and with the addition of surfactant. 

**Figure 7:**
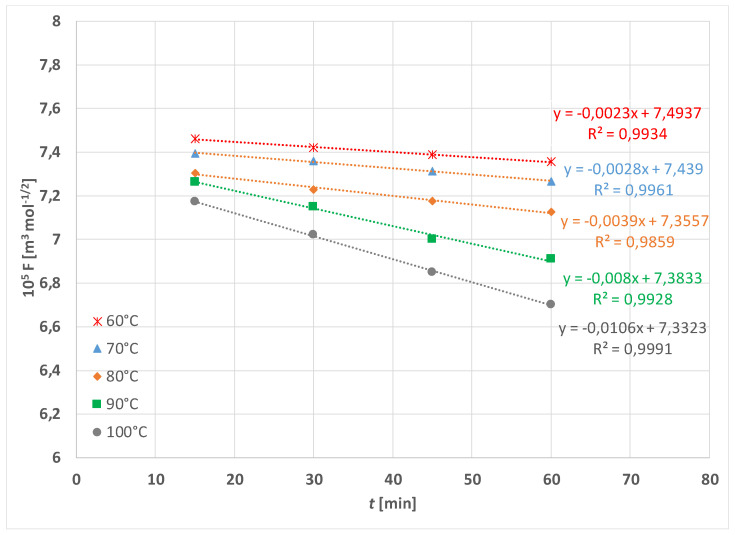
Theoretical function *F* vs. time t for hydrolysis in 1.5 mol cm^−3^ NaOH at temperature range 60 to 100 °C according to Equations (4) and (5).

**Figure 8:**
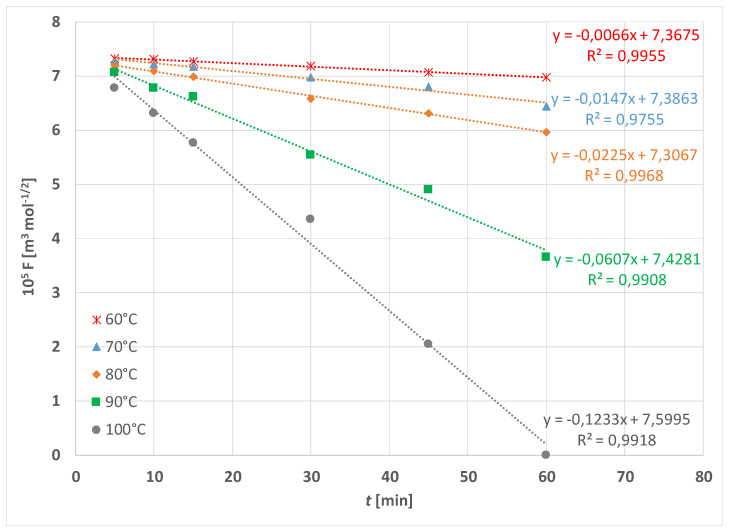
Theoretical function *F* vs. time *t* for hydrolysis in 1.5 mol cm^−3^ NaOH with addition of 1 gcm^−3^ HDTMAC at temperature range 60 to 100 °C according to Equations (4) and (5).

These results are taken into account for interpretation and used in Equations (4) and (5). According to Equation (5) the values *F* were calculated for each data and plotted vs. time. In Figure 7, the interpretation of the data from Figure 3 is given and in Figure 8 the interpretation of the data from Figure 4 is given, respectively. 

Trend lines are given of high linearity, so the rate constants were calculated from the slopes according to Equation (3) and presented in Table 4.

**Figure 9:**
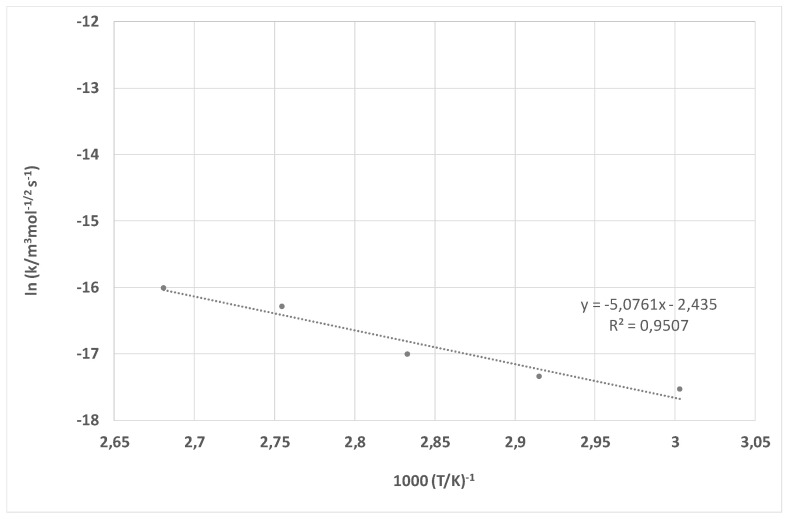
The Arrhenius plot for experiments in the 1.5 mol cm^−3^ NaOH solution.

**Figure 10:**
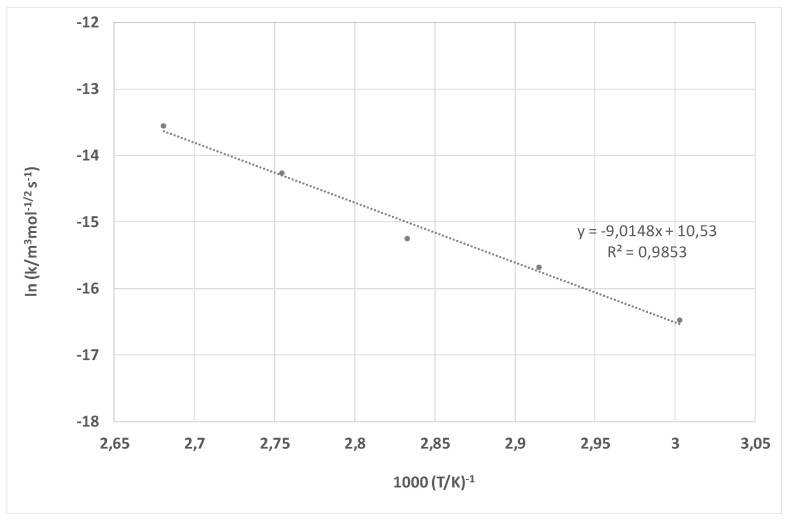
The Arrhenius plot for experiments in the 1.5 mol cm^−3^ NaOH solution in the presence of HDTMAC.

The rate constant of PET dissolution without accelerator is given as a function of temperature according to Arrhenius Equation (6) in Figure 9, and of PET dissolution with the addition of HDTMAC as an accelerator in Figure 10.

From the trend lines obtained by linear regression, the activation energy E_a_ and pre-exponential collision frequency factor *B* were calculated. It is well known that chemical reactions proceed faster at higher temperatures. However, Arrhenius Equation (6) is a result of the combination of two concepts-the activation energy and the Boltzmann distribution law and introduced the importance of the pre-exponential collision frequency factor. The pre-exponential collision frequency factor has been ignored since it is not directly involved in relating temperature and activation energy. It refers to the collision of molecules; it is the frequency of molecules that collide and with enough energy to initiate a reaction. In this paper, it was determined experimentally and proved the importance of studying it, as it varies for different reactions. For NaOH solution the activation energy of *E_a_* = 42.34 kJ mol^−1^ and pre-exponential factor *B* = 8.76 × 10^−6^ m^3^ mol^−1/2^ s^−1^. HDTMAC shows the influence on hydrolysis of polyester yielding activation energy of *E_a_* = 75.19 kJ mol^−1^ and pre-exponential factor of *B* = 3.74 × 10^5^ m^3^ mol^−1/2^ s^−1^. The PET dissolution with cationic surfactant HDTMAC, hexadecyltrimethylammonium chloride, is much faster. This reaction has higher activation energy, reduces the rate at lower temperatures, and is significantly higher than the pre-exponential factor. Comparing the activation energy achieved with HDTMAC with the ones for alkaline hydrolysis with C_14_MImBr (53.61 kJ mol^−1^), C_16_MImBr (60.76 kJ mol^−1^), and CTAB (65.31 kJ mol^−1^), it can be seen that *E_a_* for HDTMAC is even higher. This suggests that alkaline hydrolysis of polyester is more sensitive to temperature in the presence of HDTMAC and that increasing the temperature results in faster hydrolysis with the consequence of higher fiber damage and weight loss [24]. When cationic surfactant is introduced to the interface, adsorption occurs. HDTMAC has linear C16 tail and active quaternary ammonium head. Therefore, the quaternary ammonium ions adsorb to negative –OH and –COOH of PET. This adsorption is primarily electrostatic, and reverses the original negative charge of the PET. Considering the interfacial layer, this process occurs within the Stern layer at the inner Helmholtz plane [61]. Due to long hydrophobic tails, the surfactant is adsorbed head to tail with tail-tail interaction, orienting the positively charged heads from fibers toward liquid. This charge reversal facilitates attack by hydroxyl ions of NaOH and accelerate PET dissolution [10,11,12,13,14,17]. For that reason, the addition of cationic surfactants affects the transport of reactants at the PET-water interface as well as their energy state. Since the presence of HDTMAC increases the activation energy and pre-exponential collision frequency factor, it has been proven that cationic surfactant, HDTMAC, accelerates reaction compared to treatment in NaOH solutions when the surfactant was not added. The calculated model for the kinetics of polyester dissolution with the addition of HDTMAC as an accelerator enables the optimization of technological processes (i.e., the selection of suitable parameters such as temperature and processing time). This is economically favorable since, in contrast to a complex empirical approach where all four parameters (alkali concentration, amount of material, temperature, and time) have to be explored, this kinetic model allows the parameters to be calculated depending on the desired result (de-weighting or complete degradation). In addition to economic efficiency, it was also confirmed that alkaline hydrolysis of PET fabric is possible at low temperatures.

## 4. Conclusions

In this paper, the possibility of hydrolysis of the poly(ethylene-terephthalic) fibers in the fabric in a sustainable, energy-efficient process was researched. The influence of temperature on PET alkaline hydrolysis, with and without the addition of an accelerator (the cationic surfactant HDTMAC) was analyzed with respect to weight and strength loss and fiber morphology. The kinetics of PET dissolution and activation energy were determined according to the theoretical model. It has been shown that it is possible to perform hydrolysis of PET in a more environmentally friendly way compared to conventional alkaline hydrolysis (100 °C, 60 min) by alkaline hydrolysis with the addition of HDTMAC (80 °C, 10 min). This process is still not fully environmentally friendly with respect to the use of sodium hydroxide, but since good results were obtained at lower temperature and time, it is more economically and energetically acceptable compared to the conventional process, and therefore represents a more sustainable process.

## Figures and Tables

**Table 1:** Breaking force (*F*) of PET fabrics before and after hydrolysis at 60 to 100 °C.

Treatment		*F* [N]				
*t* [min]		60 °C	70 °C	80 °C	90 °C	100 °C
PET	0	638.50	638.50	638.50	638.50	638.50
NaOH	15	630.00	615.00	608.00	599.00	530.00
	30	612.00	605.00	592.00	586.50	439.00
	45	600.00	593.00	545.00	523.00	403.00
	60	589.00	571.00	503.50	448.00	371.50
NaOH + HDTMAC	5	602.00	589.00	514.50	442.50	325.00
	10	560.50	526.00	426.50	274.50	161.00
	15	507.00	452.00	350.50	166.50	30.00
	30	419.50	274.00	108.00	10.00	0.00
	45	314.50	166.50	35.00	0.00	0.00
	60	175.00	33.00	10.00	0.00	0.00

**Table 2:** Breaking elongation *(ε)* of PET fabrics before and after hydrolysis at 60 to 100 °C.

Treatment		*ε* [%]				
*t* [min]		60 °C	70 °C	80 °C	90 °C	100 °C
PET	0	34.200	34.200	34.200	34.200	34.200
NaOH	15	33.050	34.000	34.520	35.260	33.240
	30	29.910	29.910	30.000	36.924	32.700
	45	29.550	26.400	28.600	33.050	32.100
	60	29.367	29.367	26.280	32.400	31.891
NaOH + HDTMAC	5	32.009	31.926	26.132	29.050	25.781
	10	30.637	29.163	23.145	24.238	22.224
	15	27.218	25.087	23.189	18.741	18.030
	30	23.111	18.750	12.242	10.420	0.000
	45	19.772	13.180	10.376	0.000	0.000
	60	13.161	12.095	8.045	0.000	0.000

**Table 3:** Absorbency of PET fabrics before and after hydrolysis at temperatures 60 to 100 °C.

Treatment		*t* [s]				
*t* [min]		60 °C	70 °C	80 °C	90 °C	100 °C
PET	0			12.08		
NaOH	15	9.92	9.88	9.45	9.35	8.98
	30	8.12	7.99	7.80	7.05	5.29
	45	8.56	8.23	7.75	7.25	6.33
	60	9.88	9.70	7.47	9.46	7.12
NaOH + HDTMAC	5	9.23	8.90	5.81	8.22	7.59
	10	8.88	8.57	7.08	6.21	9.77
	15	8.85	8.85	7.96	7.75	7.17
	30	8.56	9.89	6.15	9.99	-
	45	8.33	7.99	6.16	-	-
	60	7.98	6.63	6.82	-	-

**Table 4:** The calculated rate constants: *k*, activation energy; *E_a_* and pre-exponential collision frequency factor *B* for PET dissolution.

Parameter	NaOH	NaOH + HDTMAC
*k*-60 °C	2.21 × 10^−8^ m^3^ mol^−1/2^ s^−1^	6.94 × 10^−8^ m^3^ mol^−1/2^ s^−1^
*k*-70 °C	2.84 × 10^−8^ m^3^ mol^−1/2^ s^−1^	1.55 × 10^−7^ m^3^ mol^−1/2^ s^−1^
*k*-80 °C	3.47 × 10^−8^ m^3^ mol^−1/2^ s^−1^	2.36 × 10^−7^ m^3^ mol^−1/2^ s^−1^
*k*-90 °C	9.46 × 10^−8^ m^3^ mol^−1/2^ s^−1^	6.38 × 10^−7^ m^3^ mol^−1/2^ s^−1^
*k*-100 °C	1.13 × 10^−7^ m^3^ mol^−1/2^ s^−1^	1.29 × 10^−6^ m^3^ mol^−1/2^ s^−1^
*E_a_*	42.34 kJ mol^−1^	75.19 kJ mol^−1^
*B*	8.76 × 10^−6^ m^3^ mol^−1/2^ s^−1^	3.74 × 10^5^ m^3^ mol^−1/2^ s^−1^

## Data Availability

Data are available in a publicly accessible repository.
